# Flexible Bronchoscopic En Bloc Cryoextraction of Endobronchial Leiomyoma Using a 1.7-mm Cryoprobe: A Case Report with One-Year Follow-Up

**DOI:** 10.3390/diagnostics15222850

**Published:** 2025-11-11

**Authors:** Chaeuk Chung, Dongil Park

**Affiliations:** 1 Division of Pulmonary, Allergy and Critical Care Medicine, Department of Internal Medicine, Chungnam National University Hospital, Daejeon 35015, Republic of Korea; universe7903@gmail.com; 2 Department of Internal Medicine, College of Medicine, Chungnam National University, Daejeon 34134, Republic of Korea

**Keywords:** endobronchial leiomyoma, central airway obstruction, flexible bronchoscopy, cryoextraction, small-caliber cryoprobe, 1.7-mm cryoprobe, airway recanalization

## Abstract

**Background and Clinical Significance:** Endobronchial leiomyoma is a rare benign tumor of the respiratory tract, accounting for less than 2% of all benign pulmonary neoplasms. Most cases have been treated surgically or with endoscopic modalities such as laser or rigid bronchoscopy-assisted cryotherapy. Flexible bronchoscopic cryoextraction has been rarely reported, typically with 2.2-mm probes. Small-caliber cryoprobes (1.1- and 1.7-mm) have been validated for diagnostic transbronchial cryobiopsy but not for therapeutic removal of leiomyoma. We report a case of complete removal of endobronchial leiomyoma using a 1.7-mm cryoprobe via flexible bronchoscopy, demonstrating full airway and physiologic recovery. **Case Presentation:** A 25-year-old never-smoking man was referred after an abnormal health-screening chest radiograph demonstrated right middle and lower lobe atelectasis. Chest CT revealed a mass obstructing the proximal bronchus intermedius. Spirometry showed reduced FEV_1_ and FVC with preserved FEV_1_/FVC ratio, consistent with central airway obstruction. Therapeutic flexible bronchoscopy (Olympus BF-1TQ290) was performed under endotracheal intubation. Initial forceps biopsies were followed by transbronchial cryobiopsy with a 1.7-mm cryoprobe, applied for five freeze–adhesion cycles. The mass detached en bloc and was retrieved without complications, resulting in complete airway recanalization and visualization of the right middle and lower lobe bronchi. Histopathology showed interlacing fascicles of bland spindle cells with cigar-shaped nuclei, positive for SMA and desmin and negative for S-100 and CD34, confirming leiomyoma. The patient was discharged the next day. At one-year follow-up, bronchoscopy and CT demonstrated no recurrence, and spirometry normalized. **Conclusions:** Reports combining flexible bronchoscopy with a 1.7-mm small-caliber cryoprobe for en bloc removal of endobronchial leiomyoma are rare. This technique may represent a minimally invasive option for selected cases, provided careful hemostatic planning and appropriate case selection.

## 1. Introduction

Endobronchial leiomyoma is an exceedingly rare benign tumor of the respiratory tract, representing less than 2% of all benign pulmonary neoplasms and an even smaller fraction of primary airway tumors overall [1]. Although leiomyomas are commonly encountered in the uterus and gastrointestinal tract, their occurrence in the lung is distinctly unusual. Within the lung, leiomyomas can be classified as parenchymal, endobronchial, or tracheal, with endobronchial tumors being particularly uncommon. Epidemiologic reports suggest that airway-based lesions occur with roughly similar frequencies in men and women; however, there appears to be a slight male predominance in cases involving the trachea or main bronchi, while parenchymal leiomyomas show a tendency to occur more frequently in females [2]. The clinical presentation is usually nonspecific, with symptoms such as cough, wheezing, or recurrent post-obstructive pneumonia, which often delay diagnosis. In some instances, endobronchial leiomyomas are discovered incidentally during bronchoscopy or chest imaging performed for unrelated reasons [3].

Diagnosis relies on a combination of imaging and endoscopic evaluation, with definitive confirmation requiring histopathology. Radiologic findings are not pathognomonic, frequently showing an intraluminal obstructing lesion or atelectasis distal to the site of involvement. Bronchoscopic inspection typically reveals a smooth, polypoid or lobulated mass protruding into the airway lumen. Histologically, leiomyomas are composed of spindle-shaped smooth muscle cells arranged in interlacing fascicles, with low mitotic activity and absence of atypia. Immunohistochemistry is positive for smooth muscle actin and desmin, while negative for cytokeratin, aiding in the distinction from other spindle cell tumors [3,4].

Therapeutic strategies for endobronchial leiomyoma vary according to tumor size, base width, and degree of airway obstruction. Historically, surgical resection—including lobectomy or sleeve resection—was the standard of care, especially for lesions causing near-complete obstruction or associated with distal parenchymal damage [3]. With advances in interventional bronchoscopy, however, less invasive techniques have gained traction. Rigid bronchoscopy enables mechanical debulking and has been combined with adjunctive modalities such as Nd:YAG laser, electrocautery snaring, and argon plasma coagulation to achieve complete resection [5]. These methods provide effective palliation and in many cases obviate the need for thoracic surgery, but their availability is often limited to specialized centers, and rigid bronchoscopy requires general anesthesia.

Cryotherapy has emerged as another bronchoscopic option. The principle of cryotherapy is based on the Joule–Thomson effect, wherein rapid gas expansion generates extreme cold, leading to tissue freezing, intracellular ice crystal formation, and subsequent necrosis [6]. Cryoablation was first introduced for managing malignant airway obstruction and was later adapted for benign tumors such as leiomyoma. Early experiences mainly utilized rigid bronchoscopes and conventional 1.9–2.4 mm cryoprobes, which permitted mechanical extraction of large tissue fragments [3,5]. However, rigid bronchoscopy is not always feasible, especially in patients with comorbidities or in centers where only flexible bronchoscopy is available. The recent development of small-caliber cryoprobes has significantly expanded therapeutic possibilities. The 1.1-mm ultrathin cryoprobe, designed for use with a guide sheath, has been validated in transbronchial cryobiopsy for peripheral pulmonary lesions. In a large series, its application was associated with a diagnostic yield exceeding 90%, surpassing that of conventional forceps biopsy, with manageable complication rates [7]. This probe enables access to distal airways through thin bronchoscopes, offering excellent flexibility while acquiring sizable, artifact-free tissue samples. Similarly, the 1.7-mm cryoprobe, thinner than conventional devices, maintains adequate tensile strength for tissue removal while providing improved maneuverability in tortuous bronchi [8]. These technological advances have been instrumental in broadening cryobiopsy and cryoextraction applications beyond malignancy to include diverse benign conditions.

Previous reports have demonstrated the diagnostic value of small-caliber cryoprobes in diverse benign and infectious pulmonary diseases [7,9]. Collectively, these studies highlight the potential versatility of cryotechnology beyond malignant disease.

Nevertheless, therapeutic cryoextraction of endobronchial leiomyoma using flexible bronchoscopy and small-caliber probes has not previously been described. While rigid bronchoscope-based cryoresection has been reported, the use of flexible cryoprobes in this specific setting remains unexplored. Given that leiomyomas are firm, encapsulated lesions with broad bases, their removal poses challenges, and the ability of small-caliber probes to achieve complete resection without surgical backup has been uncertain. The present report addresses this knowledge gap.

We describe the first case of complete removal of an endobronchial leiomyoma using a 1.7-mm small-caliber cryoprobe introduced via flexible bronchoscopy. The procedure was performed safely, without major complications, and resulted in full restoration of airway patency. Post-procedurally, objective improvement in pulmonary function testing was observed, with normalization of spirometric indices.

## 2. Case Presentation

**Figure 1:**
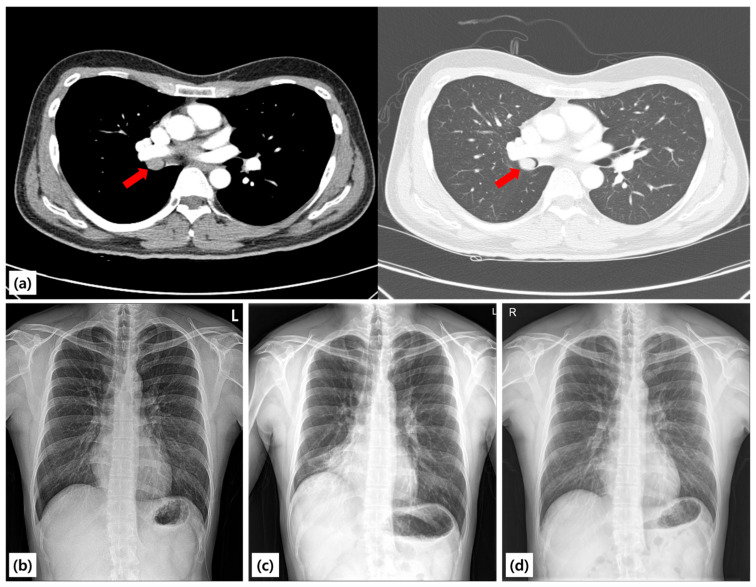
Chest radiographs and CT findings. (**a**) Pre-procedure chest CT scan showing a mass completely obstructing the proximal bronchus intermedius with distal atelectasis (red arrow). (**b**) Pre-procedure chest radiograph demonstrating volume loss of the right middle and lower lobes, manifested by elevation of the right hemidiaphragm and increased opacity in the right lower lung field, consistent with atelectasis. (**c**) Chest radiograph obtained two years earlier showing normal findings. (**d**) Post-procedure chest radiograph showing normalization of lung volume and resolution of atelectasis.

A 25-year-old never-smoking man with no comorbidities was referred to our hospital after an abnormal chest radiograph obtained during a routine occupational health screening earlier in the year. The film demonstrated right lung volume loss and an ill-defined opacity. A subsequent chest CT scan revealed a well-circumscribed endoluminal mass arising within the proximal right bronchus intermedius, causing near-complete luminal obstruction and associated distal atelectasis of the right middle and lower lobes (Figure 1). A chest radiograph taken in 2021 was entirely normal, suggesting that the lesion developed within the following three years. A chest radiograph taken in 2021 was entirely normal, suggesting that the lesion developed within the following three years. The patient denied cough, hemoptysis, chest pain, weight loss, fever, or recurrent respiratory infections. He reported only occasional exertional chest tightness during vigorous exercise, which he had not previously sought evaluation for. There was no family history of lung disease, tuberculosis, or malignancy.

**Figure 2:**
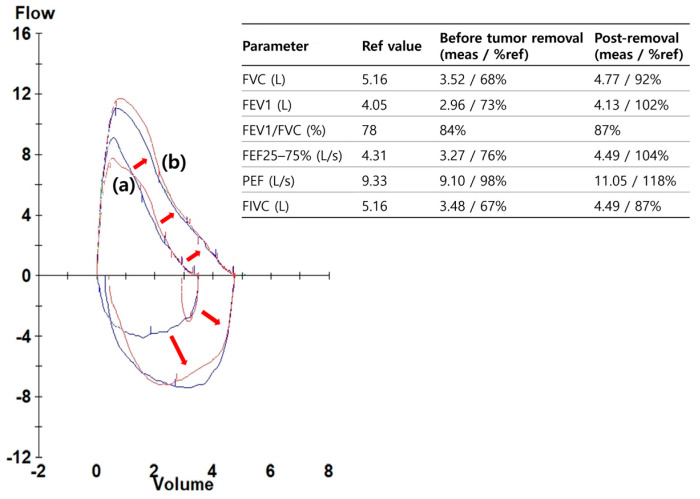
Spirometry before and after tumor removal. (**a**) Baseline spirometry at presentation showed reduced FEV_1_ and FVC with a preserved FEV_1_/FVC ratio, consistent with central airway obstruction. (**b**) Spirometry performed after tumor removal demonstrated normalization of airflow and volumes, with red arrows indicating the marked improvement of expiratory flow after relief of the obstruction.

On admission, the patient was in good general health. He was afebrile, with stable vital signs and normal oxygen saturation on room air. Chest examination revealed reduced breath sounds over the right lower hemithorax, consistent with distal airway occlusion, but there was no wheeze or stridor. The remainder of the systemic examination was unremarkable. Laboratory studies including complete blood count, electrolytes, and inflammatory markers were within normal limits. Baseline spirometry demonstrated reductions in both FEV_1_ and FVC with preservation of the FEV_1_/FVC ratio, a pattern compatible with central airway obstruction (Figure 2).

### 2.1. Procedure

**Figure 3:**
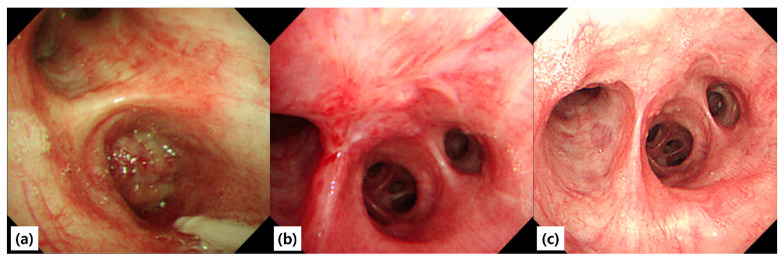
Bronchoscopic findings before and after tumor removal. (**a**) Pre-procedure bronchoscopic view showing a mass completely occluding the proximal bronchus intermedius. The base of the lesion was attached to the anterior wall between the right middle lobe (RML) and right lower lobe (RLL) orifices. The surface was nodular, with abnormally developed vessels; however, there was no spontaneous bleeding or touch bleeding. (**b**) One-month follow-up after removal, showing mild fibrosis at the previous attachment site between the RML and RLL orifices. (**c**) One-year follow-up bronchoscopy demonstrating no recurrence.

Given the radiologic evidence of an obstructing lesion and the physiologic impairment, the patient underwent therapeutic bronchoscopy under moderate sedation (conscious sedation). Airway control was secured with an 8.5-mm endotracheal tube. An Olympus BF-1TQ290 therapeutic bronchoscope (outer diameter 5.9 mm, working channel 3.0 mm) was introduced to the right bronchus intermedius. Bronchoscopic inspection revealed a smooth-surfaced, polypoid mass arising from the proximal right bronchus intermedius and completely occluding the lumen (Figure 3). The mucosa appeared intact, with no ulceration, necrosis, or vascular engorgement, findings consistent with a benign etiology.

As a precaution, a 13-mm hemostatic balloon catheter was placed in the proximal right bronchus intermedius and inflated with 2.3 mL saline to confirm effective tamponade capability in case of hemorrhage. Four transbronchial forceps biopsies were first obtained using 1.8-mm forceps (ENDO FLEX GmbH, Wesel, Germany) for histopathologic sampling. Thereafter, transbronchial cryobiopsy was performed with a 1.7-mm small-caliber cryoprobe (Erbe Elektromedizin, Tübingen, Germany). Five consecutive freeze–adhesion cycles of 5–7 s each were applied, using carbon dioxide (CO_2_) as the cryogen at approximately 35 bar working pressure, with careful withdrawal under direct visualization. Balloon tamponade was pre-tested with 2.3 mL saline to ensure full luminal occlusion and kept ready throughout the procedure. During the fifth application, the entire mass detached en bloc, remained firmly adherent to the cryoprobe, and was extracted through the bronchoscope. Following extubation, the specimen was retrieved from the oropharynx while still frozen to the probe tip.

Inspection after removal demonstrated complete recanalization of the right bronchus intermedius, with unobstructed visualization of the right middle lobe orifice and all segmental branches of the right lower lobe. Residual tissue at the tumor base was carefully ablated by additional forceps and cryo-debulking. Minor oozing was noted at the resection site but ceased spontaneously without balloon tamponade, topical hemostatic agents, or cautery. No active bleeding, airway compromise, or peri-procedural complications occurred. The total procedure time was approximately 45 min, and the patient remained hemodynamically stable throughout.

### 2.2. Pathology and Follow Up

**Figure 4:**
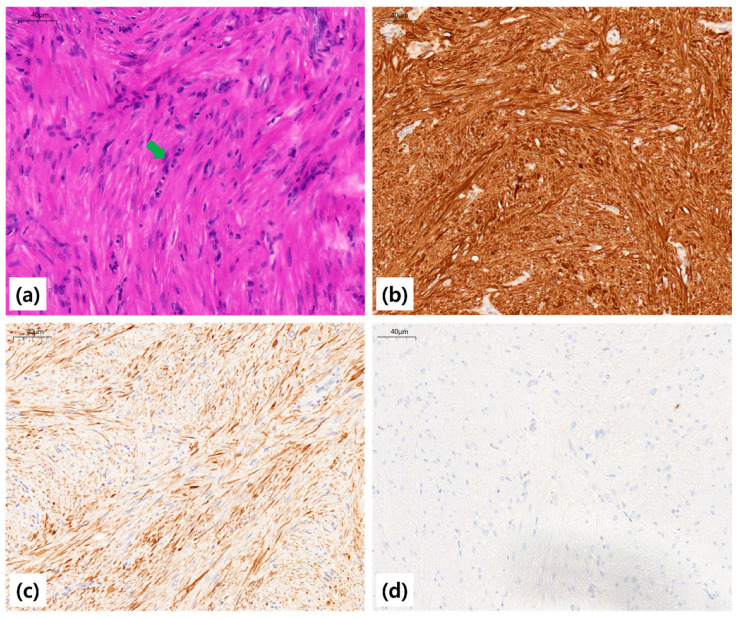
Pathologic findings of the en bloc resected mass. (**a**) Histopathology showing interlacing fascicles of bland spindle cells with cigar-shaped nuclei (green arrow), without atypical mitoses or necrosis, consistent with leiomyoma (H&E stain, ×40). Immunohistochemistry demonstrated diffuse positivity for (**b**) smooth muscle actin (SMA) and (**c**) desmin, with (**d**) negative staining for cytokeratin (CK), confirming smooth-muscle origin.

Grossly, the extracted mass measured 1.9 cm × 1.3 cm and was rubbery, well-circumscribed, and whitish. Histopathologic examination revealed interlacing fascicles of bland spindle cells with cigar-shaped nuclei, without atypical mitoses, necrosis, or cellular atypia, consistent with leiomyoma. Immunohistochemical staining demonstrated diffuse positivity for smooth muscle actin (SMA) and desmin, and negative staining for cytokeratin (CK), confirming smooth muscle origin (Figure 4).

The patient was observed overnight in the hospital. He had no hemoptysis, chest discomfort, or respiratory distress, and was discharged uneventfully the following day. Oral antibiotics and inhaled bronchodilators were not prescribed, as there was no evidence of infection or post-procedural bronchospasm. At one-week outpatient follow-up, he reported resolution of exertional symptoms, and chest radiography showed re-expansion of the right middle and lower lobes. Spirometry demonstrated normalization of FEV_1_ and FVC values, with complete resolution of the obstructive physiology (Figure 2). Baseline: FEV_1_ = 2.31 L (64% predicted), FVC = 2.72 L (68%), and FEV_1_/FVC = 0.85; Post-procedure: FEV_1_ = 3.65 L (101%), FVC = 3.82 L (98%), and FEV_1_/FVC = 0.96.

At three months, six months, and one year, surveillance bronchoscopy and chest CT scans were performed. All examinations demonstrated a widely patent right bronchus intermedius, with no evidence of residual or recurrent tumor (Figure 3). Spirometry remained normal, and the patient resumed unrestricted physical activity without limitation.

## 3. Discussion

Endobronchial leiomyoma is a rare benign tumor of the tracheobronchial tree that may mimic more common airway diseases until significant airway obstruction occurs. Because the clinical manifestations are nonspecific—most often cough, dyspnea, wheeze, or recurrent pneumonia—patients are frequently misdiagnosed with asthma, chronic bronchitis, or post-infectious airway disease before imaging or bronchoscopy reveals the obstructing lesion. Prior series have suggested a balanced sex distribution for airway lesions, whereas parenchymal leiomyomas tend to show female predominance [2,3,10,11]. The lesion in our patient arose from the proximal right bronchus intermedius, leading to complete obstruction with distal atelectasis and an obstructive ventilatory defect demonstrated on baseline spirometry. The physiologic correlation, with reduced FEV_1_ and FVC but preserved ratio, underscores the importance of pulmonary function testing in central airway lesions where classical obstructive patterns may not be evident.

Therapeutic choice depends on multiple factors, including the anatomic location of the tumor, stalk morphology (pedunculated vs. broad-based), degree of vascularity, and available expertise. Historically, surgery—ranging from lobectomy to sleeve resection—was the definitive treatment, particularly in cases with large lesions or distal parenchymal damage [3,4]. However, surgical resection carries considerable morbidity, especially in young, otherwise healthy patients, and entails loss of functional lung tissue for a benign process. For this reason, less invasive bronchoscopic modalities were explored. Heat-based technologies such as Nd:YAG laser, electrocautery snare, and argon plasma coagulation have all been used successfully to resect or ablate airway leiomyomas [3,4,5]. These methods provide rapid debulking, but thermal damage can obscure histopathology, limit tissue preservation for diagnosis, and in some cases increase the risk of airway wall injury or post-procedural stenosis.

Cryotherapy represents an alternative endoscopic modality that avoids these limitations. The mechanism, based on the Joule–Thomson effect, rapidly freezes tissue at the probe–tumor interface, forming ice crystals that disrupt cellular membranes and microvasculature, ultimately leading to necrosis and detachment. Unlike thermal modalities, cryoresection preserves tissue architecture, allowing accurate histopathologic diagnosis, which is essential in distinguishing leiomyoma from leiomyosarcoma, schwannoma, or carcinoid tumors. Early clinical experience was largely confined to rigid bronchoscopy with conventional 1.9–2.4 mm cryoprobes, permitting en bloc resection of bulky tumors [1,5,6]. Rigid bronchoscopy, however, requires general anesthesia and specialized expertise, limiting availability.

This case demonstrates that a 1.7-mm small-caliber cryoprobe used via flexible bronchoscopy under moderate sedation can achieve en bloc removal of a proximally located bronchus intermedius leiomyoma with full restoration of airway patency. This purely flexible approach differs from previous reports using rigid bronchoscopy or larger probes. This approach is noteworthy for several reasons. First, the smaller probe size provides greater flexibility, allowing easier navigation into angulated bronchi compared with conventional cryoprobes. Second, repeated freeze–adhesion cycles provide sufficient traction to extract a firm, encapsulated tumor without causing significant mucosal tearing or uncontrolled bleeding. Third, flexible bronchoscopy under moderate sedation broadens the applicability of the technique in centers without ready access to rigid bronchoscopy and reduces the anesthetic risks for patients.

Several procedural considerations were critical to the success in this case. Pre-emptive hemostatic planning was undertaken with placement of a 13-mm balloon catheter, confirming complete occlusion of the bronchus intermedius and providing immediate contingency in case of hemorrhage. This step is especially important for benign tumors where vascularity can be unpredictable, and catastrophic bleeding, although rare, can be life-threatening. The use of repeated freeze–adhesion cycles allowed gradual traction and minimized mechanical tearing. Finally, meticulous inspection and base clean-up after extraction were performed to reduce the risk of residual tumor tissue, which could serve as a nidus for regrowth. Together, these measures ensured both technical and clinical success, with normalization of spirometry and sustained airway patency at one year.

This case complements the emerging literature on the diagnostic applications of small-caliber cryoprobes. The 1.1-mm ultrathin cryoprobe has been validated for transbronchial cryobiopsy of peripheral pulmonary lesions, demonstrating improved diagnostic yield compared with forceps [7,8]. The present case extends this paradigm, demonstrating that the slightly larger 1.7-mm probe is not only a diagnostic tool but also a therapeutic instrument capable of resecting central airway tumors. Importantly, this was achieved via flexible bronchoscopy, which is more widely available than rigid techniques.

Limitations of this report include its single-case design and lack of comparative data. While the procedure was safe and effective in this instance, generalizability requires caution. Not all leiomyomas are amenable to flexible cryoextraction; broad-based or highly vascular tumors may still necessitate rigid bronchoscopy or surgical resection. Furthermore, although one-year follow-up bronchoscopy and CT demonstrated no recurrence, longer observation is required to establish durability. Future studies should aim to systematically evaluate outcomes of small-caliber cryoprobe resection in benign airway tumors, comparing efficacy, safety, and recurrence rates with conventional methods. Prospective registries could provide valuable insights into patient selection, technical refinements, and complication profiles.

This case illustrates the potential utility of small-caliber cryoprobes in interventional pulmonology, particularly for selected benign airway tumors where flexible approaches may be feasible. By combining effective tumor removal with preservation of histologic integrity and minimal morbidity, flexible bronchoscopy with a 1.7-mm cryoprobe may represent a viable alternative to more invasive procedures in selected patients. The objective normalization of lung function after tumor removal further illustrates the clinical significance of airway recanalization, beyond the technical achievement of endoscopic clearance.

## 4. Conclusions

Endobronchial leiomyoma can be managed safely and effectively with flexible bronchoscopy using a 1.7-mm small-caliber cryoprobe, when appropriate case selection and hemostatic preparedness are ensured. In addition to technical success, the objective normalization of spirometry demonstrates the clinical benefit of airway recanalization.

## Data Availability

The data presented in this case report are available on reasonable request from the corresponding author. The data are not publicly available due to patient privacy.

## Abbreviations

The following abbreviations are used in this manuscript:

BI	Bronchus intermedius
CXR	Chest X-ray
CT	Computed tomography
FEV_1_	Forced expiratory volume in 1 s
FVC	Forced vital capacity
PEF	Peak expiratory flow
FEF_25–75%_	Forced expiratory flow at 25–75% of FVC
FIVC	Forced inspiratory vital capacity
SMA	Smooth muscle actin
IHC	Immunohistochemistry
